# EMDIA Case Series—Effective Medication Therapy Management (MTM) for Diabetes Type 2 Patients—A Proof of Concept Study

**DOI:** 10.3390/pharmacy9030137

**Published:** 2021-08-09

**Authors:** Maira Anna Deters, Emina Obarcanin, Holger Schwender, Stephanie Läer

**Affiliations:** 1 Institute of Clinical Pharmacy and Pharmacotherapy, Heinrich Heine University, 40225 Duesseldorf, Germany; emina.obarcanin@hhu.de (E.O.); stephanie.laeer@hhu.de (S.L.); 2 Mathematical Institute, Heinrich Heine University, 40225 Duesseldorf, Germany; holger.schwender@hhu.de

**Keywords:** diabetes, pharmaceutical care, community practice

## Abstract

**Background**: A 2016 meta-analysis of pharmaceutical care for patients with diabetes mellitus showed that the following four components were most effective: (a) individual goal setting, (b) sending feedback to the physician, (c) reviewing the medication, and (d) reviewing blood glucose measurements. **Methods:** To formulate a hypothesis regarding the effect of these four pharmaceutical care components on glycemic control in patients with diabetes mellitus and the feasibility of these components in practice. Ten patients with type 2 diabetes were included in the case series and received medication therapy management over four months. **Results:** The four care components were feasible in everyday practice and could be implemented within one patient visit. The average visits were 49 and 28 min at the beginning and end of the study, respectively. The glycated hemoglobin values did not change over the study period, though the fasting blood glucose decreased from 142 to 120 mg/dl, and the number of unsolved drug-related problems decreased from 6.9 to 1.9 per patient by the study end. **Conclusions:** This case series supports the hypothesis that community pharmacists can implement structured pharmaceutical care in everyday pharmacy practice for patients with type 2 diabetes mellitus.

## 1. Introduction

The global prevalence of diabetes has increased in recent decades. In 2017, 58 million people were living with diabetes in Europe [1,2,3]. Although the rate of diabetes complications has decreased, patients with diabetes are still at higher risk for micro-and macrovascular complications compared to healthy persons [2,4]. Multiple randomized controlled trials (RCT) have shown that community pharmacists’ interventions can improve glycemic control in patients with diabetes mellitus [5,6,7,8,9,10,11,12,13,14,15]. Improvements in glycemic control can reduce the risk of atherosclerotic cardiovascular diseases and the occurrence of acute and long-term diabetes complications, such as severe hypoglycemia and microalbuminuria [16,17].

A 2016 meta-analysis of pharmaceutical care components provided by community pharmacists and their impact on glycemic control showed that some intervention components were more effective than others. Four of the 32 analyzed pharmaceutical care components for patients with diabetes mellitus proved remarkably useful. These were individual goal setting, sending feedback to the physician, reviewing the medication, and blood glucose measurements [18]. We selected the four most effective pharmaceutical care components due to the practicality of implementing these measures into community pharmacy practice and considering the time constraints in a community pharmacy setting. To the best of our knowledge, no study has investigated whether these four components combined were effective in practice. Most related studies have integrated more than four pharmaceutical care components, and the included elements differ between studies [18]. RCTs have a strictly controlled study setting and methodology and, therefore, high internal validity; however, the generalizability of their results is limited compared with results from real-world studies [19]. Thus, the current study aimed to formulate a hypothesis regarding the effect and practicability of conducting the four selected pharmaceutical care components in a community pharmacy.

### Objectives

The primary objective of this case series was to formulate a hypothesis about the feasibility of implementing the four mentioned pharmaceutical care components in practice. The secondary objective of this case series was to formulate a hypothesis about the effectiveness of implementing these four components in a community pharmacy setting within 60-min patient visits on different before-defined parameters: the number of drug-related problems (DRP), the medication appropriateness index (MAI), well-being index, glycated hemoglobin (HbA1c), and fasting blood glucose (FBG) values.

## 2. Materials and Methods

### 2.1. Trial Design and Participants

The EMDIA case series is a small-scale, preliminary, and qualitative pilot study with ten diabetes patients with a realistic first estimation of the expected effect and possible hurdles of implementing the main intervention. The results from this case series will serve as a basis for further planning and calculations, such as case number analysis and later implementation of further hypothesis-testing studies. Therefore, there is no calculation of sample size for these case series. We included 10 patients who had a confirmed diagnosis of diabetes mellitus, were older than 12 years, signed the informed consent form, had an HbA1c value above 7.5%, had at least four chronic diseases, had to intake medications at least 12 times daily, or had potential or manifest DRPs. The inclusion criteria were selected according to the current American Diabetes Association guidelines and the German Bundesapothekerkammer medication analysis guideline for quality assurance [20,21]. The patient data were analyzed per protocol; therefore, patients were excluded if they withdrew their informed consent or did not complete all four patient visits. Analyzing the data per protocol can confound the results. Per protocol analysis was undertaken anyway to ensure the needed amount of patient data. All patients were recruited and enrolled in one German community pharmacy in North Rhine Westphalia with face-to-face patient interviews. This study was approved by the ethics committee of Heinrich Heine University (ethical approval code: 2018-325-ProspDEuA, approval date: 25 March 2019).

### 2.2. Intervention Design

**Figure 1:**
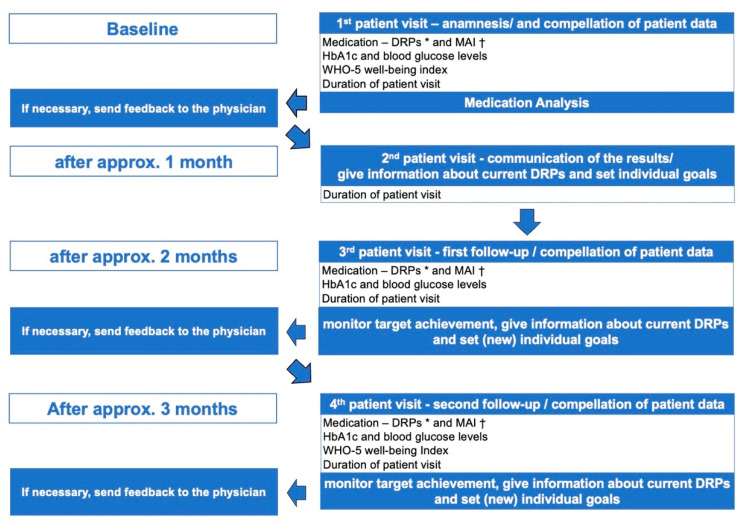
The design and chronological sequence of the case series. * DRP: drug-related problems. † MAI: medication appropriateness index.

After signing the informed consent, the pharmacist recorded the medication history; collected relevant patient data, such as HbA1c, FBG levels, and current medication; and reviewed the blood glucose recordings, where available, at baseline (see Figure 1). The patients brought their self-recorded data, blood glucose measurements, and medication history to each patient visit. The blood glucose data were recorded by self-measurement of blood glucose (SMBG). Afterward, 2 to 4 weeks after the first patient visit at baseline, the pharmacist informed the patients about potential DRPs related to their medication and set individual goals with the patient. If deemed necessary, the pharmacist sent feedback to the physician directly or indirectly (via the patient). The pharmacist gave the patient information about the current medication and checked the patient’s medication knowledge if needed. The first and second follow-up visits were conducted approximately two and three months after baseline to check whether the individual goals were achieved. In those follow-up visits we collected all relevant data such as HbA1c, FBG levels, and current medication. The WHO-5 well-being index was documented at baseline and patient visit four. If deemed necessary, the pharmacist sent repeated feedback to the physician. All interviews, medication reviews, analyses of patient data, and correspondence with the physicians were provided by one pharmacist, trained in the performance of medication reviews and medication therapy management (MTM). For this case series, the definition of the German Pharmacy Operation Regulations (ApBetrO) for MTM and medication reviews was used. MTM is a pharmaceutical activity in which the patient’s entire medication—including self-medication—is repeatedly analyzed to improve drug therapy safety and compliance by identifying and resolving DRPs. The Pharmaceutical Care Network Europe (PCNE) for medication reviews defines the medication review as a structured evaluation of a patient’s medicines to optimize medicine use and improve health outcomes. This entails detecting DRPs and recommending interventions [22]. The medication review is a central component of MTM and, if provided separately from MTM, the medication is only analyzed once. Patient data were documented according to the SOAP (subjective patient information, objective data, assessment of the data, and plan) note [23]. The individual goals were mutually set with the patient according to the SMART criteria (**s**pecific, **m**easurable, **a**ttainable, **r**ealistic, and **t**ime-based).

### 2.3. Outcomes

The primary objective of the case series was to formulate a hypothesis on the feasibility of implementing the four pharmaceutical care components mentioned before in practice. Therefore, the duration of each patient visit was documented. A total limit of 60 min per patient visit was set as realistic. In addition, the total time of each patient interview was recorded and documented. The secondary objective of the case series was to formulate a hypothesis on the effectiveness of implementing the four components in everyday practice. To develop a hypothesis about the effectiveness and successful implementation of the medication review, we identified and classified the current DRPs, according to the PCNE [24]. The PCNE classification differentiates between currently existing (manifest) and possible occurring (potential) DRPs. The results of the interventions to solve the DRPs are divided into (1) unsolved—the DRP is still completely existing, (2) partially solved—parts of the DRP are solved, (3) solved—the DRP no longer exists, or (4) DRPs with the unknown result, for example, the required laboratory values are not yet available. The MAI was determined according to Hanlon et al. and Samsa et al. [25,26]. If deemed necessary, the physician received the results of the medication reviews and relevant feedback. For evaluating the benefit of reviewing patients’ blood glucose measurements, patients’ current glycemic control, measured by HbA1c and FBG, was recorded. Mutually set individual goals can significantly impact the four pharmaceutical care components and can influence patients’ well-being. Hence, the well-being index was determined by using a German translation of the WHO-5 questionnaire [27]. Two pharmacists analyzed patient data and discussed the results of each medication review. The transmission of all data and the results were checked using the four-eye principle.

### 2.4. Statistical Analysis

To formulate a hypothesis regarding the effect of this structured MTM on the different endpoints, average values and standard deviations of all visits were compared before and after the intervention. Missing values were marked as not available (n.a.). To test whether the data is normally distributed, the Shapiro–Wilk test was used. The Wilcoxon signed-rank test was used to assess statistical significance between the different patient visits of these not normally distributed, non-parametric values. This test is applicable even when the sample size is small. A significance level of α 0.05 was chosen a priori. All statistical analyses were performed using Microsoft Excel for Mac 2019 version 16.29.1 and RStudio for Mac version 1.4.1717 [28].

## 3. Results

### 3.1. Summary-Relevant Patient Data

All of the included patients (four female and six male) had a confirmed diagnosis of type 2 diabetes mellitus. One patient was included in the case series because of an HbA1c value greater than 7.5%. Other patients had potential DRPs, such as strong daytime tiredness, hyperhidrosis, edema, or neuropathy pain. On average, patients were 70.70 ± 11.71 years old and, at baseline, had 6.0 ± 2.0 different disease states, including type 2 diabetes mellitus. The most common disease states at baseline were hypertension (*n* = 9), hyperlipidemia (*n* = 7), and chronic pain (*n* = 5). According to the patients’ statements, two patients had neuropathy and diabetic foot syndrome, and one patient had diabetic retinopathy. The average HbA1c value decreased slightly from 7.04 ± 0.90% at baseline to 7.00 ± 0.61% at the end of the case series (see Table 1). A larger, non-significant reduction was observed in the FBG levels during the case series—from 141.86 ± 32.03 mg/dL to 119.63 ± 18.95 mg/dL—while the number of unsolved DRPs was reduced significantly from 6.90 ± 2.60 at baseline to 1.89 ± 1.90 at the second follow-up (*p*-value < 0.003). The patients’ MAI values varied between 0 and 38 at baseline, with an average value of 19.1 ± 13.24. The index average decreased to 6.40 ± 8.88 by the end of the case series (*p*-value = 0.007). The WHO-5 Well-Being Index of all included patients increased from 17.10 ± 6.62 to 20.40 ± 5.83 points throughout the study.

### 3.2. Results Primary Objective—Feasibility of Implementing Four Effective Pharmaceutical Care Components

The four patients’ visits (baseline, communication of the results, and first and second follow-up visits) lasted up to 60 min. The baseline visit that included patients’ medication history documentation was the longest, with a mean duration of 48.70 min. The time of the visits varied from 14 to 60 min depending on the number of medications, the number of diseases, frequency of blood glucose measurements, and potential DRPs. The average duration of the first and second follow-up visits were 30.50 ± 10.19 min and 27.9 ± 9.57 min, respectively. All patients showed up for all four patient visits, and no patient dropped out prematurely.

### 3.3. Results Secondary Objective—Effects of the Four Pharmaceutical Care Components on Relevant Parameters

**Figure 2:**
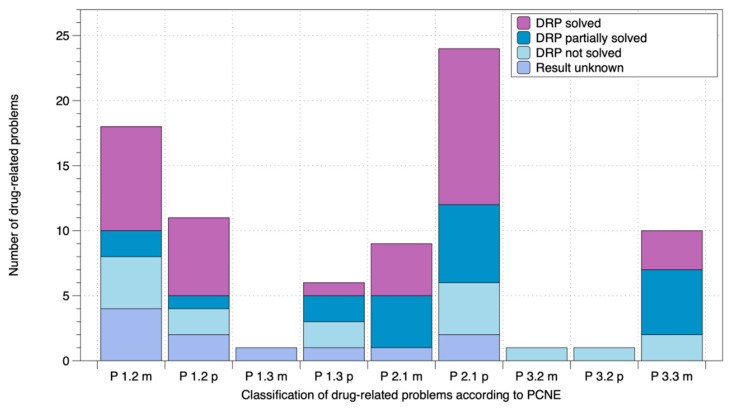
Drug-related problems of the EMDIA patients classified according to PCNE. DRPs: drug-related problems; PCNE: Pharmaceutical Care Network Europe; m: manifest; p: potential; P 1.2 effect of drug treatment not optimal; P 1.3 untreated symptoms or indication; P 2.1 adverse drug event (possibly) occurring; P 3.2 unnecessary drug treatment; P 3.3 unclear problem/complaint. Further clarification necessary.

The secondary objective of this case series was to formulate a hypothesis about the effectiveness of implementing these four components in everyday practice. To formulate a hypothesis about the effectiveness and successful implementation of the medication review, the current DRPs were identified and classified, and the MAI was determined. However, 6.90 ± 2.60 manifest or potential unsolved DRPs were identified at the beginning of the study. The most common problems were adverse drug events (24 potential and 7 manifest) and a non-optimal effect of drug treatment (11 potential and 12 manifest). The distribution of all DRPs is shown in Figure 2. After the last patient visit, approximately 45% of all DRPs were solved, and 20% were partially solved. In about 10% of the cases, the result of addressing the DRPs was not yet available.

Over the whole study period, each patient had an average of 8.2 ± 3.4 DRPs; at the end of the case series, approximately half of all potential and manifest DRPs could be solved. However, only 10% of all insulin-related DRPs were solved at the second follow-up visit; mainly, the insulin-related DRPs were solved partially (see Table 2). Still, solely 10% of insulin-related DRPs were unsolved at the study end (lowest rate). Due to pharmacist intervention, a higher rate of DRPs related to oral antidiabetics intake could be solved (62.5%). In comparison, the highest rate of unsolved DRPs was associated with residual medication, such as antihypertension medication, and just 48.4% of these DRPs were solved.

In many cases, the patients with diabetes mellitus had issues with a suboptimal effect of oral antidiabetics and/or insulin; mainly, patients had elevated or fluctuating blood glucose levels. Looking at the remaining non-antidiabetic medication, the most common was the (possible) occurrence of adverse drug events. Hypoglycemia was the most frequent adverse drug event of oral antidiabetics and insulin. The patients’ MAI values varied between 0 and 38 at baseline, with an average value of 19.1 ± 13.24. The index average decreased significantly to 6.40 ± 8.88 (*p*-value = 0.007) by the end of the case series. Thus, the MAI of most patients was relatively low, which indicated that the medication was appropriate.

For formulating a hypothesis about the benefit of reviewing patients’ blood glucose measurements, patients’ current glycemic control, measured by HbA1c and FBG, was recorded. Both HbA1c and FBG values decreased slightly, non-significantly during the study. At baseline, the average HbA1c was 7.04 ± 0.90% and decreased to 7.00 ± 0.61% at the end of the case series. The FBG levels decreased more steeply than the HbA1c values—from 141.86 ± 32.03 mg/dL to 119.63 ± 18.95 mg/dL during the case series. The review of patients SMBG data revealed that three patients experienced recurrent non-severe hypoglycemic and hyperglycemic episodes; one of these patients also had nocturnal hypoglycemia at baseline. One additional patient was identified to have poorly controlled diabetes with constant hyperglycemia.

Setting individual goals, sending feedback to the physician, and solving impairing DRPs can impact the patients’ current well-being index. Throughout the study, the WHO-5 Well-Being Index of all included patients increased significantly from 17.10 ± 6.62 to 20.40 ± 5.83 points (*p*-value = 0.02). At baseline, only two patients had fewer than 13 points, indicating that these patients had untreated depression [29,30]. One of these patients underwent professional psychological treatment but had problems adhering to the prescribed antidepressant because of its side effects. After changing the antidepressant drug, the WHO-5 Well-Being Index for this patient increased to 18. The other patient had a low well-being index score due to substantial physical restrictions. The antihypertensive medication used by this patient caused deterioration in their diagnosed sleep apnea and resulted in daytime sleepiness. Unfortunately, a change in the antihypertensive medication had no positive effect on this patient’s sleep apnea and daytime sleepiness; therefore, their WHO-5 Well-Being Index score remained less than 13.

## 4. Discussion

### 4.1. Discussion Primary Objective—Feasibility of Implementing Four Effective Pharmaceutical Care Components

Our case series provides a proof of concept that the four most effective pharmaceutical care components identified in a 2016 meta-analysis [18] are feasible for implementation in everyday pharmacy practice. The pharmacist in this study was able to conduct (1) a medication review including the identification of DRPs and determination of the MAI based on scientific evidence [24,25,26], (2) set individual goals with the patient according to the SMART criteria [31], and (3) send feedback to the physician according to the SOAP note [23,32] by using a structured form and review the blood glucose measurements within 60 min. Of course, this hypothesis that it is feasible to conduct these four effective pharmaceutical care components within 60-min patient visits in practice could also be tested as part of a hypothesis-testing study.

Other qualitative studies (case reports or analysis of pharmacist’s interviews) have evaluated the use of a special tool for implementation or the general process of implementation of pharmaceutical care in practice [33,34,35]. A study by Feletto et al. investigated the use of a research-based change-management tool in practice and its impact on the implementation of pharmaceutical care. Different influencing factors (external, internal, and individual) and barriers for implementation were identified. Other influencing external factors were, for example, the need for professional support or the remuneration of providing this service. Of course, individual factors, such as motivation to implement this research-based management tool, were identified as relevant [33]. In the German health care system, the provision of a medication review or continuous MTM is not paid by health insurance. Therefore, remuneration is a limiting factor, and the duration of all visits is a relevant influencing factor for the implementation of pharmaceutical care, especially medication reviews and MTM.

The primary objective of the analysis by Moullin et al. was to investigate the process of implementing pharmaceutical care in Australian pharmacies in practice and assess relevant influencing factors. This study identified five influences in this implementation process: direction and impetus, internal communication, community fit, staffing, and support. Each of these can positively or negatively influence several stages and activities (e.g., increased staff capacity has a positive influence on the implementation, but decreased staff capacity has a negative one) [34]. An influencing factor was, for example, the staff capacity in the community pharmacy: enough staff was available, so the pharmacist was not disturbed during the patient visits, and both study pharmacists (one pharmacist has a PharmD) were trained in the provision of MTM. In addition, of course, other influencing factors were also identified, such as the impetus of both pharmacists that had a positive impact on the implementation due to high personal interest in this topic and research field.

Silva et al. identified principles and theories that drove the implementation of pharmaceutical care and relevant components in the Brazilian public health system [35]. The study revealed the importance of educational processes and that we must pay attention to the current reality and needs of the situation in each health system. This coincides with our identified influencing factors and limitations, for example, remuneration of pharmaceutical care and training and education of the study pharmacists.

The intervention design of this case series was chosen based on the findings of the systematic literature research. In previous RCTs, pharmacist interventions have been performed monthly or every second month to explore the impact of pharmaceutical care on glycemic control in patients with diabetes mellitus [18]. Therefore, we chose the used time interval of nearly monthly pharmacist interventions. Our study’s average duration of patient visits was similar to that of other studies [11,12].

### 4.2. Discussion Secondary Objective—Effect of the Four Pharmaceutical Care Components on Relevant Parameters

#### 4.2.1. Effect on the Number of Unsolved DRPs and MAI

In our case series, 6.90 ± 2.60 DRPs were identified at baseline. The WestGem study was a German RCT that evaluated the effect of interprofessional MTM in an ambulatory setting on different outcomes (including DRPs) and included 142 patients with other chronic diseases. In that study, the average MAI was 29.21, and the number of identified DRPs per patient was 6.98 at baseline, similar to our findings. [36] The RCT conducted by Fornos et al., which included only patients with diabetes mellitus, identified 2.7 DRPs per patient at baseline, of which nearly 60% were solved (1.7 unsolved DRPs at study end per patient) [8]. Compared to our findings, approximately 45% of all DRPs were solved, and 20% were partially solved at the last patient visit; the number of unsolved DRPs was reduced from 6.90 ± 2.60 at baseline to 1.89 ± 1.90 at the second follow-up. Interestingly, the lowest rate of totally solved DRPs in this case series was insulin-related. This reveals that the adjustment of insulin therapy is particularly challenging for healthcare providers and patients. There are many possible reasons for this low rate of solved insulin-related DRPs in diabetes type 2 patients. Therefore, future studies should observe the percentage of resolved insulin-related DRPs and possible interactions, such as lack of adequate practical training beforehand, over a more extended period.

Many other studies that examined the effect of pharmaceutical care for a patient with diabetes mellitus on glycemic control included implementing a medication review. Still, none of these used the PCNE classification system for DRPs [9,12,14,15,37]. In our qualitative study, DRPs were classified according to the current PCNE classification (version 8.03). This DRP classification system was chosen because it is based on clear definitions according to Foppe van Mil et al. [38]; it has a hierarchical classification. Its validation has been published, and the pharmacist interventions are classified. Inferentially, the identified DRPs and the determined MAI depend on the included patient population, such as the number of diseases and the types of medications used, and the DRP classification system. Therefore, the comparability of results from other studies regarding the number of unsolved and solved DRPs is somewhat limited.

#### 4.2.2. Effect of the Four Pharmaceutical Care Components on HbA1c and FBG Values

The low HbA1c values at baseline (average 7.04%) and the short study duration (4 months) made it challenging to generate a hypothesis as to whether these four components, especially reviewing patient’s blood glucose measurements, can improve glycemic control, as measured by a reduction in HbA1c values and/or FBG levels. Previous RCTs of type 2 diabetes patients with higher baseline values have detected more considerable reductions in HbA1c by the end of the study, for example, Krass et al. (−1.0 [−0.8 to 1.3%]) and Doucette et al. (−0.27 ± 1.11%) [7,12]. Nevertheless, in the current study, the FBG levels decreased from an average of 141.86 ± 32.03 mg/dL at baseline to 119.63 ± 18.95 mg/dL at the end of the study. In the DIADEMA study (RCT with adolescent type 1 diabetes patients), the HbA1c values decreased from 9.4% to 8.9%, and the FBG levels decreased from 218 ± 67 to 200 ± 69 mg/dL in the intervention group [15,39]. A lower FBG level reduces the probability of developing atherosclerotic cardiovascular diseases, even in non-diabetic patients. On the other hand, impaired or diabetic FBG levels lead to increased risks for cardiovascular diseases and their complications [16,40].

The American Diabetes Association recommends that nonpregnant adults have an HbA1c value <7.0% [20]. In our case series, 50% of the patients were already within this target range at baseline. However, nearly all patients had problems with their diabetes medication and/or fluctuating blood glucose levels. Recent research has pointed out the limitations of the HbA1c value in that it only provides the average blood glucose level over the past 2–3 months. It does not indicate the patient’s current glucose variability (reoccurring hypoglycemia or hyperglycemia) [41]. A new additional predictor for glycemic control is the time in range (TIR), which assesses the percentage of time within the target (most used) or hypoglycemic range and, therefore, also takes the patient’s glucose variability into account [42]. Glucose variability can identify the deterioration of glycemic control more accurately than the HbA1c test can, thereby reducing the risk for developing diabetes-related microvascular complications [43,44,45]. TIR is usually applied in continuous glucose monitoring systems (CGMS), indicated in Germany only for patients with T1DM or T2DM on intensified insulin therapy or continuous subcutaneous insulin infusion at risk of severe hypoglycemia [46]. Hence, none of our study patients used a CGMS. Therefore, we only used HbA1c as a relevant marker for assessing overall blood glucose control. However, we are aware that if the assessment of HbA1c values is used in combination with reviewing data of CGMS a more accurate depiction of both acute and chronic glycemic control can be ascertained [47].

Consequently, further research is warranted to document and evaluate patient’s glucose variability by measuring the TIR. However, these case series generate the hypothesis that even patients with an “optimal glycemic control” measured by HbA1c values can benefit from additional pharmaceutical care provided by a community pharmacist. Therefore, we suggest examining this concept in a larger study (RCT, cohort, or case-control study) with a longer study duration of 6 or 12 months and include TIR as measured value. The inclusion criteria should also be adjusted: patients with HbA1c values above 7.5% and/or fluctuating blood glucose levels or at risk for severe hypoglycemia should be included in the study.

#### 4.2.3. Effect of the Four Pharmaceutical Care Components on the Well-Being Index

Our case series showed that the WHO-5 Well-Being Index could be easily used within this timeframe of 60 min in practice because of the short time needed for answering the five questions. In these case series, the index was used as a tool for assessing the emotional well-being of the included patients with diabetes mellitus. This brief questionnaire can be used as a quick, first-pass screening for depressive symptoms. The Diabetes MILES study supported a cutoff of <13 points to identify depression in patients with diabetes mellitus regardless of the diabetes type or treatment [29,30]. Of course, other questionnaires, such as the SF36, that assess the current health status of the diabetes patients might be more sensitive, but these more extensive questionnaires require more time to answer. Hence, we decided to use the WHO-5 Well-Being Index because the collection and documentation of relevant patient data should be implemented in everyday pharmacy practice.

### 4.3. Limitations

There are limitations in this case series, including the low patient number, patients’ low HbA1c values at baseline (average 7.04%), and the short study duration (4 months). Moreover, personnel costs and time constraints on more extended patient care visits can limit the implementation of MTM in regular community pharmacies. Regarding remuneration, the duration of all visits is relevant, as the patient or health insurance should consider reimbursing every minute of MTM or the total cost of the patient visit. As indicated, these results support the hypothesis that these four pharmaceutical care components can likely be implemented in everyday pharmacy practice. This hypothesis might be challenged by a randomized controlled trial further on.

## 5. Conclusions

As a proof of concept study of the four effective pharmaceutical care components, this case series allows us to hypothesize that structured MTM elements can be implemented in everyday pharmacy practice for type 2 diabetes mellitus patients. It is also hypothesized that implementing these four pharmaceutical care components can improve diabetes mellitus type 2 patients’ FBG levels, the number of unsolved DRPs, MAI, and WHO-5 Well-Being Index scores. Further research should be conducted to clarify the effect of these four components on glycemic control, especially on HbA1c values and TIR, in diabetes patients in everyday pharmacy practice.

## Figures and Tables

**Table 1:** Overview of relevant data and study endpoints.

Relevant Data and Study Endpoints	1st Patient Visit: Anamnesis	3rd Patient Visit: First Follow-Up	4th Patient Visit: Second Follow-Up
**Medication**			
Number of medications per patient	10.50 ± 3.75 *n* = 10	11.00 ± 4.37 *n* = 10	10.90 ± 4.07 *n* = 10
Unsolved DRPs ^1^ per patient	6.90 ± 2.60 *n* = 10	2.30 ± 2.11 *p*-value: <0.003 * n = 10	1.89 ± 1.90 *p*-value: <0.003 * n = 10
Solved DRPs ^1^ per patient	none	3.20 ± 1.99 *n* = 10	3.70 ± 2.45 *n* = 10
Partially solved DRPs ^1^ per patient	none	1.45 ± 2.16 *n* = 10	1.80 ± 2.30 *n* = 10
DRPs ^1^ per patient with unknown result	none	0.90 ± 1.28 *n* = 10	1.00 ± 1.41 *n* = 10
Average MAI ^2^	19.10 ± 13.24 *n* = 10	10.20 ± 9.80 *p*-value: 0.007 * n = 10	6.40 ± 8.88 *p*-value: 0.007 * n = 10
**Glycemic control**			
HbA1c value [%]	7.04 ± 0.90 *n* = 9	6.90 ± 0.56 *p*-value: 0.50 *n* = 9	7.00 ± 0.61 *p*-value: 0.78 *n* = 9
Fasting blood glucose [mg/dL]	141.86 ± 32.03 *n* = 7	147.29 ± 31.76 *p*-value: 0.85 *n* = 7	119.63 ± 18.95 *p*-value: 0.05 *n* = 8
**Other relevant measurements**			
Average WHO-5 Well-Being Index	17.10 ± 6.62 *n* = 10	no measurement	20.40 ± 5.83 *p*-value: 0.02 * n = 10
Duration of patient visits	48.70 ± 8.83 *n* = 10	30.50 ± 10.19 *n* = 10	27.90 ± 9.57 *n* = 10

Mean-values ± standard deviation; *n* = number of analyzed patients; * significant *p*-values (*p* < 0.05); ^1^ DRPs = drug-related problems classified according to PCNE; ^2^ MAI = medication appropriateness index.

**Table 2:** Number of drug-related problems of EMDIA patients sorted by the type of medication.

	Solved DRPs ^1^	Partially Solved DRPs ^1^	Unsolved DRPs ^1^	DRPs ^1^ With Unknown Result
Oral antidiabetics	0.5 ± 0.7 (62.5%)	0.1 ± 0.3 (12.5%)	0.1 ± 0.3 (12.5%)	0.1 ± 0.3 (12.5%)
Insulin	0.1 ± 0.3 (10%)	0.6 ± 1.1 (60%)	0.1 ± 0.3 (10%)	0.2 ± 0.6 (20%)
Residual medication	3.1 ± 1.9 (48.4%)	1.1 ± 1.7 (17.2%)	1.5 ± 2.0 (23.4%)	0.7 ± 1.1 (10.9%)

Mean-values ± standard deviation (percentage); ^1^ DRPs = drug-related problems classified according to PCNE.
